# Simultaneous Vascularized Lymph Node Transfer and Breast Reconstruction: A Systematic Review

**DOI:** 10.3390/jcm14051694

**Published:** 2025-03-03

**Authors:** Hamzah Almadani, Jocelyn Lu, Sara Bokhari, Christiane How-Volkman, Philip S. Brazio

**Affiliations:** 1 Division of Plastic and Reconstructive Surgery, Department of Surgery, Cedars-Sinai Medical Center, Los Angeles, CA 90211, USA; hamzah.almadani@cshs.org (H.A.); jocelyn.lu@cshs.org (J.L.); 2 College of Osteopathic Medicine of the Pacific, Western University of Health Sciences, Pomona, CA 91766, USA; sara.bokhari@westernu.edu; 3 College of Medicine, California Northstate University, Elk Grove, CA 95757, USA; christianemhv@gmail.com

**Keywords:** lymphatic reconstruction, VLNT, vascularized lymph node transfer, simultaneous

## Abstract

**Background/Objectives**: Simultaneous vascularized lymph node transfer (VLNT) and breast reconstruction is a reconstructive option that potentially addresses two adverse consequences of breast cancer treatment in the same operation. This systematic review aims to analyze the quality of data and outcomes in the current literature. **Methods**: This systematic review was performed following PRISMA guidelines. A systematic search was conducted with Google Scholar and PubMed for studies with the simultaneous intervention of VLNT and breast reconstruction. The search terms were ((diep OR pap OR expander OR implant OR breast OR msTRAM OR TRAM) AND (“vascularized lymph node”)). Studies were included if they were original articles that discussed patients who underwent simultaneous VLNT and breast reconstruction. Additional sources were identified from bibliographies. Patient characteristics, types of treatment, reconstruction, and outcome measures were collected. This review was not registered. **Results**: A total of 1969 unique English literature search results led to the inclusion of 118 studies. Further analysis was performed on 42 non-review articles, documenting a total of 772 patients. The mean patient age was 51.6 years, the mean BMI was 28, and there was a mean follow-up of 23.8 months. Discrete breast reconstruction data were given for 494 patients with 492 autologous reconstructions and 2 tissue expander-to-implant reconstructions. The most common reconstructive approach was a deep inferior epigastric flap. The most common VLNT donor site was the superficial inferior epigastric nodes, followed by superficial circumflex iliac nodes. Indications for 646 patients were for treatment and 18 were for prevention, while 108 were not specified. The mean excess volume reduction in treatment studies was 39.5%. A total of 168 complications (21.8%) were reported, with donor site seromas being the most common. Additionally, four partial and three total flap failures were reported. **Conclusions**: VLNT can be safely combined with autologous breast reconstruction for the treatment or prevention of breast cancer-related lymphedema. Future research should standardize the approach for data collection and report patient outcomes for lymphedema and immediate lymphatic reconstruction.

## 1. Introduction

Breast cancer-related lymphedema (BCRL) is a chronic, often debilitating condition characterized by the accumulation of protein-rich fluid under the skin, typically affecting the extremities. This condition results from an obstruction of lymphatic outflow and functioning due to cancer treatment. The incidence of unilateral arm lymphedema after breast cancer is estimated to be 14.9–29.8% [1]. The initial management of BCRL typically involves conservative approaches, such as complete decongestive therapy (CDT), compression garments, and manual lymphatic drainage. These measures aim to reduce fluid accumulation and alleviate symptoms such as swelling, pain, and a reduced range of motion [2,3]. However, for patients with moderate to severe or refractory cases, surgical interventions have become an increasingly viable option. Among surgical options, lymphovenous bypass and vascularized lymph node transfer (VLNT) are two advanced microsurgical techniques that have gained prominence in the last few decades [4].

VLNT was introduced as a treatment for lymphedema in the late 20th century for lower extremity lymphedema, and it has since been adapted for BCRL [5]. A VLNT is a free flap containing viable lymph nodes that is transferred to the affected area to re-establish lymphatic function. The recipient site of VLNT, whether orthotopic (proximal) and/or heterotopic (distal), has been the subject of debate [6]. Orthotopic placement relies on the lymphangiogenesis stimulated by growth factor secretion and incorporates the radical scar release of the axilla with the ease of combination with autologous breast reconstruction, potentially allowing for a single scar. Heterotopic placement relies on a “sump” mechanism of lymphatic drainage via the flap vein and favors distal placement at the antecubital fossa or wrist.

Given that breast reconstruction often accompanies breast cancer treatment, combining orthotopic VLNT with reconstruction could offer dual benefits by addressing both cosmetic and lymphatic concerns simultaneously [7]. Despite its use as a treatment for lymphedema, there is a lack of information on the outcomes of the simultaneous use of VLNT with breast reconstructive surgery to prevent or treat BCRL.

This systematic review seeks to assess the quality of existing data and examine outcomes in the current literature on VLNT performed concurrently with breast reconstruction to prevent or treat BCRL.

## 2. Methods

### 2.1. Search Strategy and Study Selection

This systematic review followed the PRISMA guidelines [8]. A comprehensive search strategy was utilized, on 16th October 2024, across two databases—PubMed and Google Scholar—to aggregate studies including VLNT in the setting of concurrent breast reconstruction. Across both databases, the following search strategy was indexed: “DIEP” OR “PAP” OR “Expander” OR “Implant” OR “Breast” OR “msTRAM” OR “TRAM” AND “Vascularized Lymph Node”. Upon initial title and abstract screening, articles were excluded if there was no relevance to breast reconstruction or axillary VLNT; if the studies were primarily non-English; or if the studies were in the form of nonclinical studies, book chapters, or abstracts. Case reports, series, and prospective and retrospective studies were included if the primary language was English and included at minimum one patient who underwent VLNT with simultaneous breast reconstruction. Partial breast reconstruction using oncoplastic techniques was not excluded. Full-text screening removed studies in which VLNT was simultaneously performed at the time of breast reconstruction. A final review of the selected articles excluded reviews from data extraction and subsequent analysis. Two independent reviewers determined the inclusion of studies in each phase of study selection. If discrepancies arose, a third independent reviewer determined inclusion. This review was not registered. The protocol was not prepared.

### 2.2. Data Extraction and Statistical Analysis

Two reviewers collected study variables in Microsoft Excel from each article. Study variables included author, title, publication year, publication type, the Oxford Centre for Evidence-Based Medicine (OCEBM) level of evidence, the number of patients, whether studies were therapeutic versus prophylactic, average patient age, average patient body mass index (BMI), breast cancer treatment classification, the presence of adjuvant chemotherapy or radiation, the type of breast reconstruction, the donor site of VLNT, axillary lymph node treatment, and the number of nodes removed, the assessment method of lymphedema pre-operatively and post-operatively, the average length of follow-up, pain scores, lymphedema excess volume reduction, excess circumference reduction, and complications. Excess volume/circumference reduction was defined as the percent reduction in the excess volume/circumference of the affected arm when compared to the normal arm:

$$\frac{\left(PAV-PUV\right)-(TAV-TUV)}{\left(PAV-PUV\right)}$$

*PAV*: pre-treatment affected arm volume, *PUV*: pre-treatment unaffected arm volume, *TAV*: post-treatment affected arm volume, *TUV*: post-treatment unaffected arm volume.

A risk of bias assessment was performed by one author using the Risk of Bias in Non-randomized Studies of Interventions tool (ROBINS-I, V2).

## 3. Results

### 3.1. Search Results

**Figure 1:**
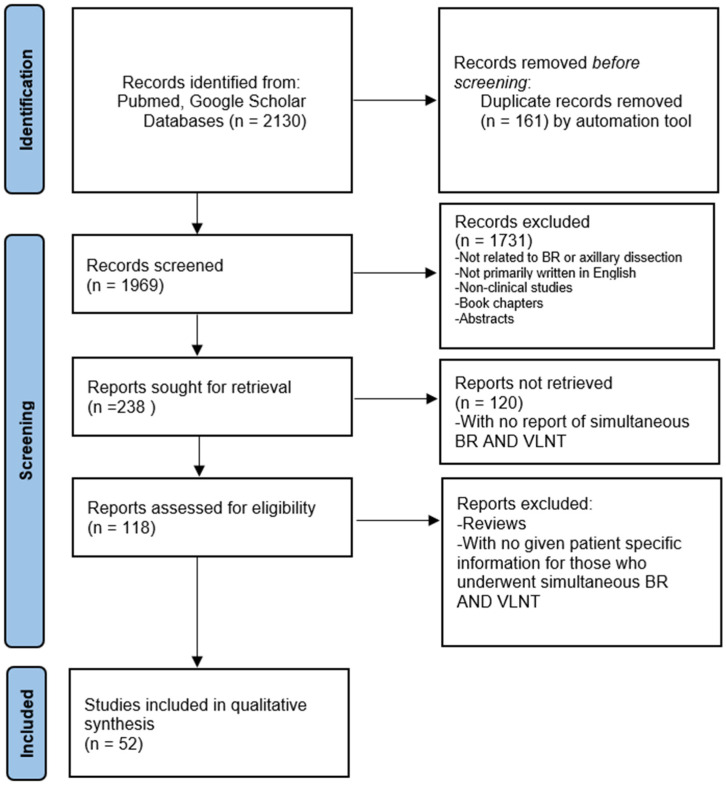
Preferred reporting items for systematic reviews and meta-analysis (PRISMA) diagram; breast reconstruction (BR) and vascularized lymph node transfer (VLNT).

Our initial search resulted in 1969 unique search results. Title and abstract screening resulted in 238 articles for full-text review, of which 118 met the eligibility criteria (Figure 1, Table 1). Studies were published between 2009 and 2024. Of the 118 articles, 66 were review articles including 42 literature review articles, 8 narrative reviews, 10 systematic reviews, and 6 systematic reviews and meta-analyses. The 52 remaining non-review articles included 1104 patients; 23 articles were retrospective cohort studies, 13 were case series, 7 were case reports, 5 were prospective cohort studies, 1 was a prospective observational study, 1 was a randomized clinical trial, 1 was a prospective randomized clinical study, and 1 compared a retrospective cohort to a prospective cohort. The mean OCEBM level of evidence was 3.66.

### 3.2. Patient Characteristics

Of the 52 non-review articles, 10 did not include patient-level data on patients undergoing simultaneous VLNT and breast reconstruction. The remaining 42 articles [3,10,11,12,13,14,15,16,17,18,19,20,21,22,23,24,25,26,27,28,29,31,32,33,36,37,38,39,41,42,43,44,45,46,47,48,49,50,51,52] included a total of 772 patients with a mean age of 51.6 years and a mean BMI of 28. The mean follow-up was 23.8 months; 18 patients underwent procedures for the prevention of lymphedema, 646 patients underwent procedures for lymphedema treatment, and indications were not specified for 108 patients.

330 patients had cancer treatments recorded. 181 patients underwent mastectomies, while 39 underwent lumpectomies; 128 patients were treated with adjuvant chemotherapy, 16 underwent neoadjuvant chemotherapy and 221 underwent radiation therapy. There were 5 patients who underwent axillary sentinel lymph node biopsy and 238 patients with axillary dissection. Two articles [27,42] with a total of 55 patients reported an average of 24.2 nodes dissected.

### 3.3. Comorbidities

Eight articles [16,27,30,33,38,45,51,52], including 255 patients, reported data on comorbidities in their patients; 30 (12%) patients were smokers, 4 (1.6%) were prior smokers, 24 (9.4%) patients were diagnosed with hypertension, 9 (3.5%) were diagnosed with diabetes, 4 (1.6%) had a history of deep vein thromboses, 3 (1.2%) had hypothyroidism and 2 (7.8%) had hyperlipidemia. One database study [45] with 75 patients reported an elevated risk of complications within 30 days in patients with increased BMI, longer operations, diabetes, smoking, hypertension, and COPD but did not include patient-specific data.

### 3.4. Breast and Lymphatic Reconstruction

Discrete breast reconstruction data were given for 494 patients; 492 reconstructions were autologous, including 418 deep inferior epigastric perforator (DIEP) flaps, 48 transverse rectus abdominis muscle (TRAM) flaps (40 muscle-sparing TRAM, 8 non-muscle-sparing), 14 latissimus dorsi (LD) flaps, 10 lateral thoracic artery (TDAP) flaps, 1 superior gluteal artery (SGAP) flap, and 1 omental flap. Two patients from one manuscript underwent two-staged, tissue expander to implant reconstruction [37]. This manuscript did not specify at what stage the lymph node transfer was completed.

Three studies, including 38 patients, described partial breast reconstruction using oncoplastic techniques simultaneously with VLNT for lumpectomy defects. Of these, 25 patients underwent lipofilling or local flaps, 10 underwent lateral thoracic artery perforator flaps, and 2 patients with segmental defects were reconstructed with DIEP free flaps; 1 segmental defect was reconstructed with greater omentum containing vascularized lymph nodes [23,25,37].

Discrete lymphatic reconstruction data were given for 670 patients. The donor site of choice for VLNT was superficial circumflex iliac artery (SCIA) nodes for 93 patients, superficial inferior epigastric artery (SIEA) nodes for 210 patients, omental lymph nodes for 74 patients, deep inferior epigastric perforator flap with attached inguinal nodes for 55 patients, lateral thoracic nodes for 21 patients, and unspecified groin lymph nodes for 65 patients. Seven articles, including 152 patients, used a mix of SIEA, SCIA, and SCIP flap pedicles but did not specify how many patients were for each donor site.

### 3.5. Simultaneous VLNT and Breast Reconstruction Outcomes

Across 22 articles, 333 out of 415 patients (80%) reported significant patient-reported improvement in BRCL symptoms, while 2 articles included 27 (6.5%) patients with no reported improvement; 48 (12%) patients reported improvement using LYMQOL, 11 (2.7%) patients reported the reduction in or cessation of garment use or therapy, and 265 (64%) reported only subjective improvement; 9 (2.2%) experienced a decrease/cessation in episodes of cellulitis.

Twenty-one articles reported an excess volume reduction. Of these, 18 articles, including 287 patients, reported a mean of 39.5% excess volume reduction. Three articles found no difference.

Nine articles reported an excess circumference reduction. Of these, four articles, including 37 patients, reported percentages, for a mean of 33.5% excess circumference reduction. Two articles reported no difference (Table 2).

### 3.6. Complications

Out of 772 total patients, 168 (21.8%) had reported complications. Donor site seromas were most common (46), followed by abdominal wound dehiscence and delayed healing (42), skin infections (15), donor site pain/numbness (14), recipient site delayed wound healing (10), and other complications (Table 3). There were 3 total flap losses (0.4%), of which 2 were chimeric LD/lateral thoracic node flaps and 1 was not specified. There were 4 reported partial flap losses (0.5%), 2 of which were chimeric DIEP/groin node flaps, a DIEP flap necrosis, and 1 DIEP/Omental VLNT (without specification of which flap suffered the partial loss). There were 4 reported cases of donor site lymphedema (0.5%).

### 3.7. Risk of Bias

**Figure 2:**
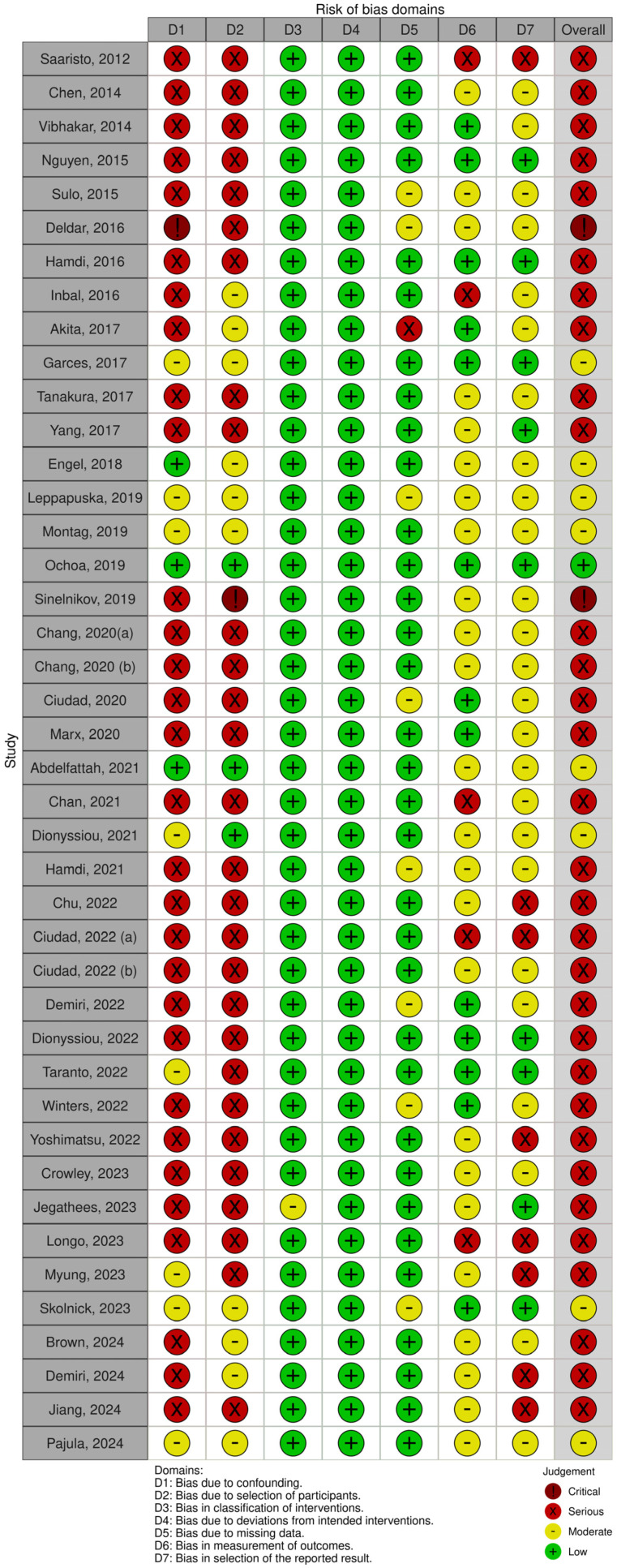
ROBINS-I assessment table [3,9,10,11,12,13,14,15,16,17,18,19,20,21,22,23,24,25,26,27,28,29,30,31,32,33,34,35,36,37,38,39,40,41,42,43,44,45,46,47,48,49].

The Robins-I tool was used to assess the quality of the aforementioned 42 analyzed articles; 2 articles were considered to be of critical risk, 31 were considered of serious risk, 8 of moderate risk, and 1 of low risk (Figure 2).

## 4. Discussion

Breast cancer is the most common non-skin cancer in women in the United States, and the incidence of breast cancer continues to increase [53]. While breast reconstruction has traditionally focused on recreating a breast mound, today’s reconstructive solutions continue to evolve to more completely restore form and function. As options for implant-based and autologous reconstructive surgeries expand, so too does the paradigm of what breast reconstruction should include. Awareness of BCRL has increased as more options for surgical treatment and prevention are offered to patients. As such, patient discussions regarding reconstruction should include options for lymphatic reconstruction along with recreating the breast mound. Many breast cancer patients require adjuvant therapy as part of multimodal treatment; therefore, the ideal reconstructive outcome must be balanced with minimizing delays in oncologic care. Simultaneous lymph node transfer has the potential to improve reconstructive outcomes without increasing the number of operations.

There is a rich literature on breast reconstruction, on VLNT, and on simultaneous lymphovenous bypass with breast reconstruction in patients with breast cancer, but most articles do not address simultaneous VLNT and breast reconstruction. The articles included in this review show promising results. Symptomatic improvements were seen in 80% of patients and limb volumes were reduced by up to 39.5%. Overall complications for both breast reconstruction and VLNT were relatively consistent with previously published studies of either surgery.

The ideal timing of breast reconstruction depends on the patient, the surgeon, and the breast cancer treatment needed. Delayed-immediate free flap reconstruction is commonly used to mitigate complications related to multiple surgeries and adjuvant therapies [54]. Similarly, performing VLNT at a stage following the mastectomy may be favored to reduce the complexity of the initial operation and improve outcomes.

The choice of donor site for VLNT must balance morbidity and ease of access with the expected effectiveness of nodes from different donor sites. Donor site complication rates range from less than 1% to 16% [55,56]. The most common complications are delayed wound healing, lymphocele, seroma, and donor site discomfort or numbness [55,56]. The most disturbing complication of VLNT is donor site lymphedema. Our systematic review found an overall 0.4% rate of iatrogenic donor site lymphedema. Surgeons performing VLNT must be well versed in lymphatic drainage pathways and lymph node anatomy. The use of reverse lymphatic mapping identifies nodes that are crucial for the drainage of a limb using technetium-99, indocyanine green, or another lymphatic dye [34,57] and allows the surgeon to avoid harvesting them and causing donor site lymphedema. To our knowledge, donor site lymphedema has never been reported when using reverse lymphatic mapping, and we recommend its use every time lymph nodes are harvested from non-omental donor sites. Omental VLNT is another safe choice, as there have been no reports of donor site lymphedema after omental harvesting. The relative effectiveness of various donor sites in treating lymphedema has been the subject of some debate [35,55], and the number of nodes transferred may affect the outcome [56]. The groin, omental, submental, supraclavicular, and lateral thoracic donor sites average three nodes [35,58,59,60], while the inguinal packet ranges from two to five lymph nodes [32]. Inguinal nodes are easily accessed during dissection for patients undergoing DIEP flap reconstruction, and omental lymph nodes may be amenable for open harvest through a DIEP harvest incision as well. Lateral thoracic nodes are potentially visualized through a mastectomy, through an axillary lymph node dissection incision, or during the dissection of an LD flap.

VLNT is a well-established treatment for lymphedema patients suffering from International Society for Lymphology stages 2–3 lymphedema, usually when no intact target channels are available for lymphovenous bypass or in patients suffering from recurrent cellulitis [3,61,62,63]. The use of VLNT for stage 0 lymphedema, on the other hand, has not been well established or studied. We identified three manuscripts [34,46,50] that used VLNT for patients with stage 0 lymphedema after axillary dissection. Ciudad et al. described simultaneous immediate breast and axillary reconstruction using DIEP flap and omental VLNT at the time of mastectomy and axillary dissection and noted no increased arm circumference and no changes in lymphography up to 3 years post-operatively [50]. In a case series of 13 patients, Brown et al. found that immediate VLNT after upper or lower extremity lymph node dissection was able to prevent increased limb volume in 12 of 13 patients, and none of the 13 patients required compression garments post-operatively with an average of 12 months follow-up [46]. The authors also point out the added benefits of replacing soft tissue at the axilla to prevent painful, potentially disabling axillary contractures, especially in those receiving radiation therapy. Yoshimatsu et al. used the DIEP flap and SCIA nodes for simultaneous breast and axillary reconstruction in four patients with stage 0 lymphedema, none of whom developed symptoms after 38 months [40].

The results of our systematic review favor the routine use of VLNT for patients who are appropriate free flap candidates and have undergone mastectomy and axillary dissection. Simultaneous breast reconstruction and VLNT seem to be effective, with overall low complication rates at both donor and recipient sites. A lack of consistency and standardized objective measurements makes it difficult to compare outcomes across different techniques and patient populations. Standardized outcome reporting, along with controlled studies of VLNT in stage 0 lymphedema patients, should be encouraged.

### Limitations

This systematic review has several limitations which based on the studies included. There is substantial heterogeneity with treatment study type, treatment regimens, population size, results reporting and follow up. This heterogeneity creates difficulty in comparing outcomes across techniques and population sizes. Given the variability of study reporting and this study being retrospective in nature, bias may have been included in the reported outcomes. The lack of prospective studies is also noteworthy. Prospective study, use of controls, and standardized analysis and reporting parameters are encouraged in future studies.

## 5. Conclusions

Early studies incorporating breast reconstruction with VLNT show promising results. Larger data sets with standardized, long-term outcome reporting are needed to compare techniques and indications. Based on the results of this review, we recommend simultaneous VLNT with breast reconstruction for any patient who has undergone axillary dissection and is a free flap candidate.

## Figures and Tables

**Table 1:** Studies describing simultaneous breast reconstruction and vascularized lymph node transfer. VLNT: vascularized lymph node transfer, BR: breast reconstruction, DIEP: deep inferior epigastric perforator, TRAM: transverse rectus abdominus muscle, TE: tissue expander, LD: latissimus dorsi, SGAP: superior gluteal artery perforator, SCIA: superior circumflex iliac artery, SIEA: superior–inferior epigastric artery, ICG: indocyanine green.

Authors	Year	Indication for VLNT	Type of Breast Reconstruction	Donor Site	N	Follow-Up (Months)	Outcome
Saaristo et al. [9]	2012	Treatment	TRAM, DIEP	SCIA	9	24	Simultaneous BR and VLNT is an optimal option for BRCL.
Chen et al. [10]	2014	Treatment	TRAM, DIEP	SCIA	10	12	BR with simultaneous VLNT and adjuvant lymphedema therapy is effective in restoring function of the breasts
Vibhakar et al. [11]	2014	Treatment	LD	Lateral Thoracic	1	2.5	Simultaneous LD flap and VLNT is an excellent solution for BR and lymphedema treatment.
Nguyen et al [12]	2015	Treatment	TRAM, DIEP	SIEA	29	11	The algorithm discussed in this paper shows promising results for simultaneous abdominal BR and VLNT.
Sulo et al. [13]	2015	Treatment	DIEP, TRAM	SCIA	21	34.8	Care must be taken when harvesting donor lymph nodes, although no clinical donor lymphedema was found in the study population.
Deldar et al. [14]	2016	Treatment	DIEP	Omentum	5	NA	Gastroepiploic lymph nodes for use of simultaneous BR and VLNT allows for less risk compared to the groin.
Hamdi et al. [15]	2016	Treatment	DIEP	SCIA	22	29.9	VLNT significantly improves the quality of life in BRCL patients.
Inbal et al. [16]	2016	Treatment	LD	Lateral Thoracic	11	6.7	LD for BR with VLNT is a viable option for treatment of BRCL.
Akita et al. [17]	2017	Treatment	DIEP	SCIA	13	13.9	The DIEP flap is a strong option for simultaneous breast reconstruction when using the SCIA as a donor site for VLNT.
Garces et al. [18]	2017	Not specified	DIEP, SGAP	NA	23	23.8	ICG is an appropriate method to determine functional lymphovenous communications following BR with VLNT.
Tanakura et al. [19]	2017	Treatment	DIEP	SIEA	1	NA	On average, 3.67 nodes exist superior to the saphenofemoral junction using multidetector CT images.
Yang et al. [20]	2017	Treatment	TRAM, DIEP	SCIA	10	12	TRAM/DIEP VLNT and BR is a safe and effective treatment for patients with post-mastectomy lymphedema.
Engel et al. [21]	2018	Treatment	DIEP	SCIA	11	15.4	BR alone does not improve BRCL, but VLNT, with or without BR significantly improved BRCL.
Leppapuska et al. [22]	2019	Treatment	TRAM, DIEP	SCIA	10	SCIA	Simultaneous operations including BR, VLT, and liposuction are better than any procedure alone.
Montag et al. [23]	2019	Treatment	DIEP	SCIA	9	18	Simultaneous BR and VLNT positively impacts patients with BRCL with no difference in relation to the position of the flap.
Ochoa et al. [24]	2019	Not specified	DIEP	DIEP	10	NA	DIEP is an appropriate lymphatic donor site for BR and VLNT.
Sinelnikov et al. [25]	2019	Treatment	Greater Omental Flap	Omentum	1	6	BR using the greater omental flap, with its high lymphatic capability, allows for correction of disrupted fluid drainage in the upper extremities.
Chang et al. [26]	2020	Treatment	DIEP	SCIA, SIEA	54	12	DIEP flap with VLNT and lymphovenous bypass may be superior to BR and VLNT alone.
Chang et al. [27]	2020	Treatment	DIEP	SCIA	38	19.1	Simultaneous BR with VLNT, with or without lymphovenous bypass, is a promising treatment for BRCL.
Ciudad et al. [28]	2020	Treatment	DIEP	Omental	6	12.8	Combined DIEP and Omental VLNT is a safe, reliable, single-staged operation for BRCL.
Marx et al. [29]	2020	Treatment	DIEP	Thoracodorsal	5	6	BR and VLNT allow for reductions in lymphedema and pain and improved quality of life.
Abdelfattah et al. [30]	2021	Treatment	DIEP	SCIA	6	30	BR with simultaneous VLNT to the axilla or forearm is effective and reliable for treatment of BRCL.
Chan et al. [31]	2021	Treatment	DIEP	SIEA	2	23	VLNT, BR, and lymphatic anastomosis together seems to be better than VLNT and BR alone.
Dionyssiou et al. [32]	2021	Treatment	DIEP, LD	SIEA, SCIA	24	36	Larger flaps consisting of a higher number of LNs were associated with improved outcomes following simultaneous BR and VLNT.
Hamdi et al. [33]	2021	Treatment	DIEP	SIEA	65	62.4	Seroma is the most likely complication of simultaneous VLNT and BR.
Chu et al. [34]	2022	Treatment	DIEP	SIEA	3	NA	Patients suffering from BRCL can be safely treated with simultaneous BR and VLNT, with or without lymphovenous bypass.
Ciudad et al. [3]	2022	Preventative	DIEP	Omentum	1	36	Simultaneous VLNT and BR allows for the possibility of the prevention of lymphedema without the risk of iatrogenic lymphedema.
Ciudad et al. [35]	2022	Treatment	DIEP	Omentum	10	NA	For advanced stages of BRCL, a combination of procedures along with BR-VLNT is needed.
Demiri et al. [36]	2022	Treatment	DIEP	SCIA	1	28	Patient reported high level of satisfaction with breast and lymphedema reconstructions.
Dionyssiou et al. [37]	2022	Treatment	TDAP, DIEP, LD, 2 Stage TE	Lateral Thoracic, Groin	69	56	VLNT with BR allows for a single surgical procedure to provide the best outcome in post-mastectomy lymphedema patients.
Taranto et al. [38]	2022	Treatment	DIEP	SIEA	32	42.5	Simultaneous DIEP and VLNT improves the quality of life of lymphedema patients and use with abdominal flap at no increased risk.
Winters et al. [39]	2022	Treatment	DIEP	DIEP	45	51.1	Simultaneous VLNT and DIEP flap breast reconstruction can cause significant improvement in BRCL patient quality of life, with or without a change in volume difference.
Yoshimatsu et al. [40]	2022	Preventative	DIEP	SCIA, SCIP	4	33.9	Use of SCIP flap from Zone 4 region in DIEP flap breast reconstruction can improve and/or prevent lymphedema without need of another donor site.
Crowley et al. [41]	2023	Treatment	TRAM, DIEP	Omentum	7	14.6	The omentum is a versatile and safe door site for simultaneous VLNT and BR.
Jegathees et al. [42]	2023	Treatment	TRAM	SCIA	1	49	Simultaneous BR and VLNT allows for a functional solution for BR with disrupted lymphatic systems.
Longo et al. [43]	2023	Treatment	DIEP	SIEA	1	3	Retrograde flow from the thoracodorsal artery is a viable option as a recipient vessel and enhances the success of DIEP flap BR VLNT.
Myung et al. [44]	2023	Treatment	TRAM, DIEP	Omentum	49	27.05	All surgical methods in the study, including VLNT with BR using the omental flap resulted in reduction in edema and improved patient satisfaction.
Skolnick et al. [45]	2023	Not specified	NA	NA	75	1	VLNT with BR for BRCL does not significantly change the risk from BR alone.
Brown et al. [46]	2024	Preventative	DIEP	Omentum	13	15.1	VLNT is a promising procedure to minimize lymphedema and contracture following extensive lymph node dissection and radiotherapy
Demiri et al. [47]	2024	Treatment	DIEP	SCIA	34	35	The algorithm proposed for DIEP and VLNT allows for “highly satisfactory” breast and lymphedema reconstruction.
Jiang et al. [48]	2024	Treatment	DIEP	SCIA	2	12	VLNT is an effective treatment for BRLC, with or without BR.
Pajula et al. [49]	2024	Treatment	DIEP	SCIA	26	12	VLNT with reverse lymphatic mapping is safe and does not increase risk of donor site lymphedema with or without BR.

**Table 2:** Simultaneous breast reconstruction and vascularized lymph node transfer outcomes.

Outcome	
**Significantly Improved Symptoms**	332 patients
Reported in	22 (52.4%) articles
**Improved Limb Volume**	
Mean Excess Volume Reduction	39.5%
Reported in	21 (50%) articles
Mean Excess Circumference reduction	33.5%
Reported in	9 (21.4%) articles
**No Change in Limb Volume**	
No Change in Excess Volume	57 Patients
Reported in	3 (7.1%) articles
No Change in Excess Circumference	2 Patients
Reported in	2 (4.8%) articles

**Table 3:** Complications following simultaneous breast reconstruction and vascularized lymph node transfer; rates per reported 772 patients.

Complication	Number (%)
Total Complications	168 (21.8%)
Donor Site Seroma	46 (6%)
Abdominal Wound Dehiscence/Delayed Wound Healing	42 (5.4%)
Skin Infections	15 (1.9%)
Donor Site Pain/Numbness	14 (1.8%)
Recipient Site Delayed Wound Healing	10 (1.3%)
Non-specified Infections	8 (1%)
Recipient Site Infection	6 (0.8%)
Donor Site Lymphedema	4 (0.5%)
Breast Flap Donor Site Hernia	4 (0.5%)
Venous Thrombosis	4 (0.5%)
Fat Necrosis	4 (0.5%)
VLNT + BR Partial Flap Failure	3 (0.4%)
Total Flap Failure	3 (0.4%)
Flap Congestion	2 (0.3%)
Skin Necrosis	2 (0.3%)
Partial Breast Flap Failure	1 (0.2%)

## Data Availability

Data are contained within the article.
